# Immunomodulatory Effects of the Cyclooxygenase Inhibitor Lornoxicam on Phenotype and Function of Camel Blood Leukocytes

**DOI:** 10.3390/ani11072023

**Published:** 2021-07-06

**Authors:** Jamal Hussen, Mahmoud Kandeel, Turke Shawaf, Abdullah I. A. Al-Mubarak, Naser A. Al-Humam, Faisal Almathen

**Affiliations:** 1 Department of Microbiology, College of Veterinary Medicine, King Faisal University, Al-Ahsa 31982, Saudi Arabia; aialmubark@kfu.edu.sa (A.I.A.A.-M.); nalhumam@kfu.edu.sa (N.A.A.-H.); 2 Department of Biomedical Sciences, College of Veterinary Medicine, King Faisal University, Al-Ahsa 31982, Saudi Arabia; mkandeel@kfu.edu.sa; 3 Department of Pharmacology, Faculty of Veterinary Medicine, Kafrelshikh University, Kafrelshikh 33516, Egypt; 4 Department of Clinical Sciences, College of Veterinary Medicine, King Faisal University, Al-Ahsa 31982, Saudi Arabia; tshawaf@kfu.edu.sa; 5 Department of Public Health, College of Veterinary Medicine, King Faisal University, Al-Ahsa 31982, Saudi Arabia; falmathen@kfu.edu.sa; 6 The Camel Research Center, King Faisal University, Al-Ahsa 31982, Saudi Arabia

**Keywords:** dromedary camel, lornoxicam, leukocytes, flow cytometry, ROS, phagocytosis, apoptosis

## Abstract

**Simple Summary:**

The present study investigated the immunomodulatory effects of the unspecific cyclooxygenase inhibitor lornoxicam on the immunophenotype and some functions of dromedary camel blood leukocytes. Intravenous injection of camels with a single dose of lornoxicam induced a significant change in the camel leukogram, which is characterized by reduced cell numbers of all leukocyte subpopulations. In vitro analysis of cell vitality revealed a pro-apoptotic effect of lornoxicam on camel leukocytes, which may be responsible for the lornoxicam-induced leukocytopenia in vivo. Functional ex vivo and in vitro analysis of the key antimicrobial functions, phagocytosis and ROS production indicates inhibitory effects of lornoxicam on the antimicrobial capacity of the blood phagocytes, monocytes and neutrophils. Furthermore, lornoxicam induced an anti-inflammatory phenotype of monocytes, characterized by reduced expression of major histocompatibility complex (MHC) class II molecules and increased expression of CD163 molecules. The present study identified for the first time inhibitory effects of the COX-inhibitor lornoxicam on some phenotypic and functional properties of camel blood immune cells and recommends considering these effects when using lornoxicam in camel medicine.

**Abstract:**

(1) Background: Lornoxicam is a nonsteroidal anti-inflammatory drug (NSAID) with analgesic, antiphlogistic and antipyretic effects. The improved tolerance of lornoxicam due to the relatively shorter elimination half-life in comparison to other members of the oxicams may favor its application in the management of pain and inflammation in race dromedary camels. There are no studies conducted yet on the immunomodulatory or immunotoxilogic effect of lornoxicam in camels. Therefore, the current study aimed to evaluate the immunomodulatory effects of the cyclooxygenase inhibitor lornoxicam on some phenotypic and functional properties of camel blood leukocytes; (2) Methods: Using flow cytometry, blood leukocyte composition, monocyte phenotype, and antimicrobial functions of neutrophils and monocytes were analyzed ex vivo after a single dose injection with lornoxicam. In addition, the effect of in vitro incubation of camel blood with lornoxicam on leukocyte cell vitality and antimicrobial functions were evaluated; (3) Results: The injection of camels with a single dose of lornoxicam resulted in a significant change in their leukogram with reduced numbers of total leukocytes, neutrophils, eosinophils, monocytes, and lymphocytes. Within the lymphocyte population, the numbers of CD4^+^ T cells, γδ T cells, and B cells decreased significantly in blood after injection of camels with lornoxicam. In addition, injection of lornoxicam resulted in decreased abundance of major histocompatibility complex (MHC) class II molecules and increased abundance of the scavenger receptor CD163 on blood monocytes, indicating an anti-inflammatory phenotype of monocytes. Functionally, administration of lornoxicam decreased the capacity of camel neutrophils and monocytes to uptake bacteria and to produce reactive oxygen species (ROS) after bacterial stimulation. Similarly, the in vitro whole blood incubation with lornoxicam resulted in reduced phagocytosis and ROS production activity of the camel blood phagocytes. Flow cytometric analysis of cell vitality, including cell necrosis and apoptosis, revealed a pro-apoptotic effect of lornoxicam on camel leukocytes; (4) Conclusions: Lornoxicam administration, at the dose and intervals utilized herein, induces significant changes in the phenotype and function of camel blood leukocytes. The reduced cell numbers of all studied leukocyte subpopulations in lornoxicam-treated camels, which seems to be a result of enhanced cell apoptosis, indicates an inhibitory effect rather than a modulatory effect of lornoxicam on the camel immune system, which need to be considered when using lornoxicam in camel medicine.

## 1. Introduction

The importance of pain management in animals has increased significantly in recent decades [1,2,3,4,5]. Nonsteroidal anti-inflammatory drugs (NSAIDs) are the most frequently used analgesic drugs in veterinary practice, especially when pain is associated with inflammation such as in muscular and orthopedic injuries [6]. The mode of action of NSAID depends primarily on the inhibition of cyclooxygenases, the enzymes that promote the synthesis of prostaglandins which are significantly increased in inflammation [7,8,9]. Depending on their structure, NSAIDs can be classified into two main groups, including carboxylic acid and enolic acid derivatives. Oxicams, including piroxicam, isoxicam, meloxicam, tenoxicam, and lornoxicam, are a class of NSAIDs structurally related to the enolic acid subgroup [10]. Although oxicams are mainly used in human medicine, some of the members were also introduced to veterinary medicine [11].

Lornoxicam (chlortenoxicam) is a type of oxicams with analgesic, anti-inflammatory, and antipyretic properties mainly mediated by the inhibition of prostaglandins, with a better tolerance due to the relatively shorter elimination half-life in comparison to other members of the oxicams class [12,13,14,15,16]. In addition, the inhibitory potential of lornoxicam on in vitro prostaglandin synthesis in rat neutrophils was found 100-fold more than tenoxicam and prevented the arachidonic acid-induced lethality in mice more efficiently than indomethacin or piroxicam [12,15,17]. In human monocytes and macrophages stimulated with lipopolysaccharide (LPS), lornoxicam induced a higher potent anti-inflammatory effect than other NSAD, including piroxicam, diclofenac, ibuprofen, ketorolac, and naproxen [17].

In humans, a dose of lornoxicam is rapidly absorbed from the gastro-intestinal system with a plasma half-life of 4 h after dosing [18]. After catabolization, lornoxicam is converted to the 5′-hydroxy-metabolite, which is inert in pharmacological tests [15]. The oxidative metabolism of lornoxicam is made by cytochrome CYP2C9 (member of the family of cytochrome P450) [19]. The pharmacokinetics of lornoxicam is influenced by the CYP2C9 polymorphism. The presence of the CYP2C9*3 allele reduced the oral clearance of lornoxicam [19]. Furthermore, the CYP2C9*1/*13 genotype has been linked to a considerable decrease in lornoxicam metabolism [20].

The shorter half-life of lornoxicam makes its application appear advantageous in the management of pain and inflammation in racing dromedary camels. However, oxicams, like all other NSAIDs, have shown the potential for several adverse effects that should be considered [21]. To the best of our knowledge, there are no studies conducted yet on the immunomodulatory or immunotoxologic effect of lornoxicam in camels. The aim of the present work was, therefore, to evaluate the effect of a single injection dose with lornoxicam on some phenotypic and functional characteristics of camel blood leukocytes, and to investigate the in vitro impact of lornoxicam on leukocyte viability and antimicrobial functions.

## 2. Materials and Methods

### 2.1. Animals Handling, Drug Administration, and Blood Collection

Six adult female Arabian camels (Camelus dromedarius) aged between 9 and 12 years (median age 10.3 ± 1) were included in the present study. The camels were reared at the research farm of the Camel Research Center at King Faisal University (Al-Hofuf, Saudi Arabia). The animals were housed in a conventional free-stall pen. All animals were fed on hay and barley in addition to a mineral supplement. Water was available ad libitum. The animals were checked for apparent health conditions and normal health indicators. For all animals, a general clinical examination was performed to exclude animals with fever, infectious diseases such as mastitis, metritis, or respiratory infections, or inflammatory disorders such as joint and bone inflammation. Using blood samples collected before treatment, leukocyte count and composition were also estimated, and only animals with a normal (non-inflammatory, non-stressed) leukogram were included in the study. The ethics committee of King Faisal University approved all animal procedures (approval no. KFU-REC/2020-03-02). Lornoxicam (trade name is Xefo) was obtained from Nycomed, Zürich, Switzerland. Camels were injected with a single dose of lornoxicam (0.13 mg/kg by the intravenous route). Six hours after injection, blood samples collection was performed by venipuncture of the *vena jugularis externa* into blood collection tubes containing the anticoagulant EDTA. The animals are kept only for experimental purposes, and there was no clinical indication for using lornoxicam.

### 2.2. In Vitro Incubation of Whole Blood with Lornoxicam

The in vitro whole blood assay was performed as previously described with modifications [22]. Camel EDTA blood (1 mL) was mixed (1:2) with the cell culture medium RPMI 1640 in sterile 12 × 75 mm tubes (BD Biosciences, Heidelberg, Germany). The samples were then incubated (37 °C, 5% CO2) with 0.1, 1, 10 or 100 µmol/L lornoxicam (final concentration) for 6 h. After incubation, leukocytes were separated by lysing erythrocytes. Separated leukocytes were used for cell viability, phagocytosis, and ROS analysis studies.

### 2.3. Separation of Blood Leukocytes

For blood leukocytes separation, erythrocytes were removed by hypotonic lysis [23]. Briefly, camel blood was diluted with PBS (1:2) and the diluted samples were centrifuged for 10 min at 1000× *g* and 4 °C. For removing erythrocytes, 5 mL of distilled water were added to the cell pellet for 20 s followed by the addition of a similar volume of double-concentrated PBS. In the case of non-complete erythrolysis, the procedure was repeated until having a clear white pellet of leukocytes.

### 2.4. Monoclonal Antibodies

The primary and secondary antibodies used for cell labeling in the present study are shown in Table 1.

### 2.5. Membrane Immunofluorescence and Flow Cytometry

The expression levels of several cell markers were analyzed using membrane immune cell labeling and flow cytometry [24]. Isolated camel blood leukocytes were suspended in PBS supplemented with bovine serum albumin (5 g/L) and NaN_3_ (0.1 g/L) (MIF buffer; membrane immunofluorescence buffer) at 5 × 10^6^ cells/mL. Leukocyte suspension (100 µL containing 4 × 10^5^ cells) was incubated with primary monoclonal antibodies (mAbs) against the following cluster of differentiation (CD) antigens: CD4, WC-1, CD14, CD163, and major histocompatibility complex (MHC) class II molecules [25]. To remove the unbound antibodies, the cells were washed twice with MIF buffer (by addition of 150 µL buffer followed by centrifugation for 3 min at 300× *g* and 4 °C). To detect the cells labeled with mouse primary antibodies, fluorochrome-conjugated secondary antibodies directed against mouse isotypes (IgM, IgG1, IgG2a; Invitrogen; Schwerte, Germany) were added to the cells. Control set-ups were stained with isotype control antibodies. Finally, labeled cells were washed twice with MIF buffer and analyzed using flow cytometry (Accurie C6 flow cytometer, BD Biosciences). After the measurement of 100.000 total leukocytes, data were analyzed using the CFlow Software (BD Biosciences; Heidelberg, Germany).

### 2.6. Analysis of Cell Viability and Apoptosis

Cell necrosis was measured by the dye exclusion assay [26]. Briefly, camel leukocytes were labeled with propidium iodide (PI) at a final concentration of 2 µg/mL (Calbiochem, Germany) and PI-fluorescence was measured by flow cytometry (detected in FL-3). PI-positive dead cells with permeable cell membranes were distinguished from PI-negative live cells. For the measurement of cell apoptosis, separated leukocytes (100µL in RPMI-1640 cell culture medium) were incubated with JC-1 (5,5′,6,6′-tetrachloro-1,1′,3,3′-tetraethylbenzimidazolcarbocyanine iodide) in a 96-well microtiter plate [27,28,29]. JC-1 solution (100µL of 2μmol/L final concentration) was added to the cells in each well for 15 min (5% CO2) at 37 °C. Finally, labeled cells were washed twice with PBS, suspended in 200μL PBS, and analyzed on the Acuri C6 flow cytometer (BD Biosciences). Apoptotic cells with JC-1 monomers (increased green fluorescence) were distinguished from normal non-apoptotic cells with orange JC-1 aggregations (increased orange fluorescence in FL-2). Hydrogen peroxide (H2O2; 180 uM)) has been used to establish a positive control of apoptosis.

### 2.7. Phagocytosis Assay

The phagocytosis activity was measured after the incubation of camel leukocytes with heat-killed bacteria. *S. aureus* bacteria (Pansorbin^®^, Calbiochem, Merck, Nottingham, UK) were labeled with fluoresceinisothiocyanate labeling kit (FITC, Sigma-Aldrich, Germany) according to the instructions of the manufacturer. Labeled *S. aureus* (30 bacteria/cell) were added to separated leukocytes (100 µL RPMI medium containing 4 × 10^5^ cells) for 30 min at 37 °C. After two washing rounds (by addition of 150 µL RPMI medium followed by centrifugation for 3 min at 300× *g* and 4 °C), the samples were analyzed on the flow cytometer. The percentage of green fluorescing cells among the total cell population was used to indicate the phagocytic activity of monocytes and neutrophils, while the mean fluorescence intensity (MFI) of positive cells was used to indicate the number of bacteria ingested by each cell.

### 2.8. Reactive Oxygen Species (ROS) Generation Assay

Monocytes and neutrophils ROS production was measured using the labeling with dihydrorhodamine (DHR)-123 as previously described [30]. Leukocytes (1×10^6^/well) were incubated in RPMI medium with *S. aureus* with the addition of DHR-123 (500 ng/mL) for 20 min at 37 °C. After two washes, the labeled cells were analyzed by flow cytometry. ROS generation was determined using the MFI of DHR-positive cells after the acquisition of 10.000 monocytes or neutrophils.

### 2.9. Statistical Analyses

The statistical software program GraphPad Prism (GraphPad software version 5, GraphPad Software, San Diego, CA, USA) was used to perform statistical analysis. The results were expressed as mean ± standard error of the mean (SEM). Data normal distribution was explored using the Kolmogorov Smirnov test. The student *t*-test was used to test the difference between the means of the analyzed parameters before and after administration of lornoxicam. The differences between the means of more than two groups were compared using the one-way ANOVA. Bonferroni’s multiple comparison test has been performed to compare all pairs of means. A difference between the means was considered statistically significant if the p-value was less than 0.05.

## 3. Results

### 3.1. The Impact of Lornoxicam on the Leukogram of Dromedary Camels

**Figure 1:**
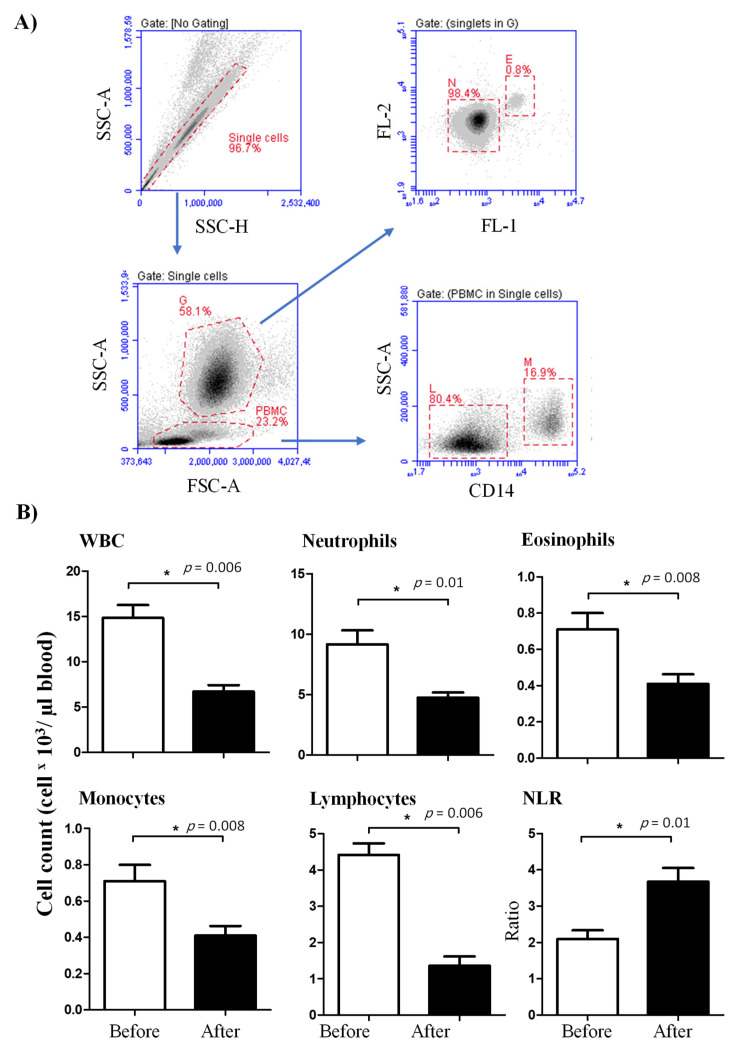
Ex vivo analysis of the leukogram of lornoxicam-treated camels. (**A**) Flow cytometric gating strategy for the analysis of leukocyte subpopulations. Doublets were excluded in a side scatter height (SSC-H) against SSC-Aria (SSC-A). In a SSC-A against forward scatter (FSC)-A dot plot, granulocytes (G) and peripheral blood mononuclear cells (PBMC) were identified based on their scatter characteristics. Based on their different autofluorescence intensities in the green FL-1 channel, eosinophils (E) were distinguished from neutrophils (N) [25]. Monocytes (M) and lymphocytes (L) were identified as CD14-positive and CD14-negative PBMC, respectively. (**B**) Whole white blood cells (WBC) were counted under the microscope after erythrolysis using Turk solution. The cell numbers of leukocyte populations were estimated by multiplication their percentages in blood leukocytes with the absolute number of WBC. Mean cell numbers and standard error of the mean were presented for all leukocyte subsets before and after treatment. Differences between the means were considered significant (*) if *p* < 0.05.

The total cell number of leukocytes (WBC) in blood was significantly (*p* < 0.05) reduced after injection of camels with lornoxicam (6.7 ± 0.7) in comparison to WBC count before treatment (14.8 ± 1.4 × 10^3^/µL). The differential flow cytometric analysis of the main leukocyte subpopulations (Figure 1A) revealed significantly lower numbers of neutrophils (4.7 ± 0.4 versus 9.1 ± 1.2 × 10^3^/µL before treatment), eosinophils (0.4 ± 0.1 versus 0.7 ± 0.1 × 10^3^/µL before treatment), monocytes (0.4 ± 0.1 versus 0.7 ± 0.1 × 10^3^/µL before treatment), and lymphocytes (1.4 ± 0.3 versus 4.4 ± 0.3 × 10^3^/µL before treatment) in blood of camels after treatment with lornoxicam than before treatment (Figure 1B) (*p* < 0.05). The treatment induced a decrease in neutrophils numbers resulting in significantly higher values of neutrophil to lymphocyte ratio (NLR) in blood after treatment with lornoxicam than before treatment (Figure 1B). The mean leukocyte viability in our experiments was above 94% with no impact of treatment with lornoxicam on the percentage of viable leukocytes (PI-negative cells).

### 3.2. Lymphocyte Composition in the Blood of Lornoxicam-Injected Camels

**Figure 2:**
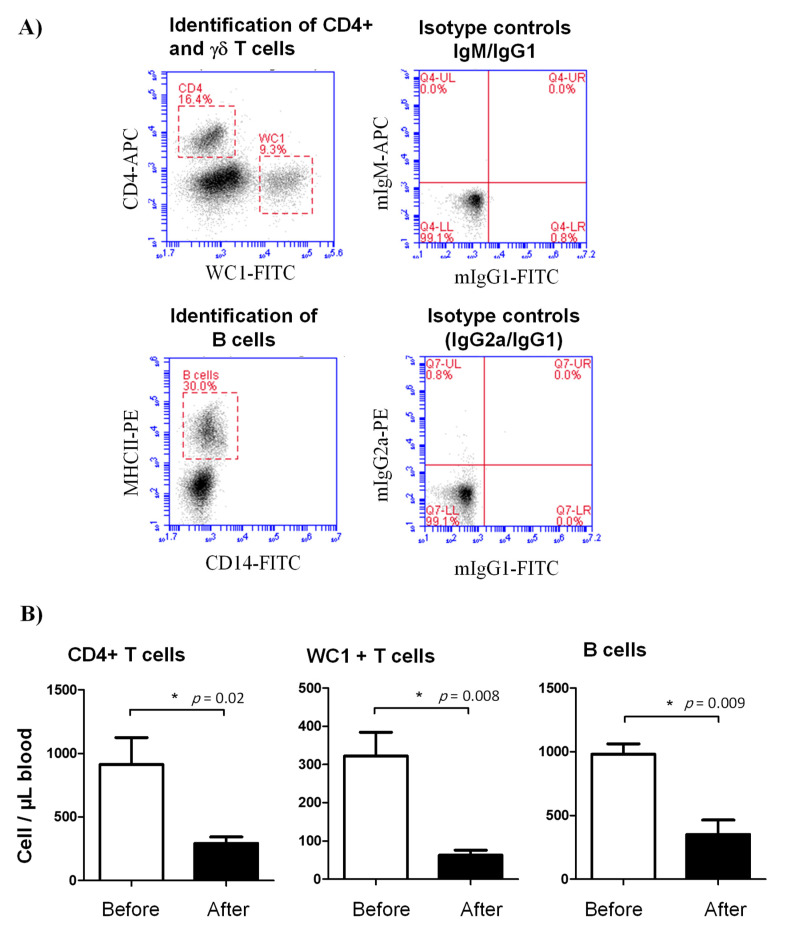
Lymphocyte composition in the blood of lornoxicam-treated animals. (**A**) Flow cytometric gating strategy for the identification of selected lymphocyte subsets in camel blood. After gating on lymphocytes within the mononuclear cell population in a SSC-A/FSC-A dot plot, CD4+ T cells and γδ T cells were identified based on their single-staining with CD4 and WC-1, respectively. In a CD14 against MHC-II dot plot, camel B cells were identified as MHC-II+CD14- cells. The cell numbers of camel B cells, CD4+ T cells, and γδ T cells were estimated by multiplication of their percentages in blood lymphocytes with the absolute number of lymphocytes. Mean cell numbers and standard error of the mean were presented for all subsets before and after treatment. (**B**) Differences between the means were considered significant (*) if *p* < 0.05.

In comparison to their cell counts before the injection with lornoxicam, the number of CD4+ T cells (293.3 ± 50.2 versus 913.7 ± 210.4 cell/µL before treatment), γδ T cells (62.9 ± 13.0 versus 322.4 ± 61.9 cell/µL before treatment) and B cells (352.4 ± 112.1 versus 982.5 ± 80.2 cell/µL before treatment) in the blood of camels decreased significantly (*p* < 0.05) after treatment with lornoxicam (Figure 2A,B).

### 3.3. Lornoxicam Modulates the Phenotype of Blood Monocytes

**Figure 3:**
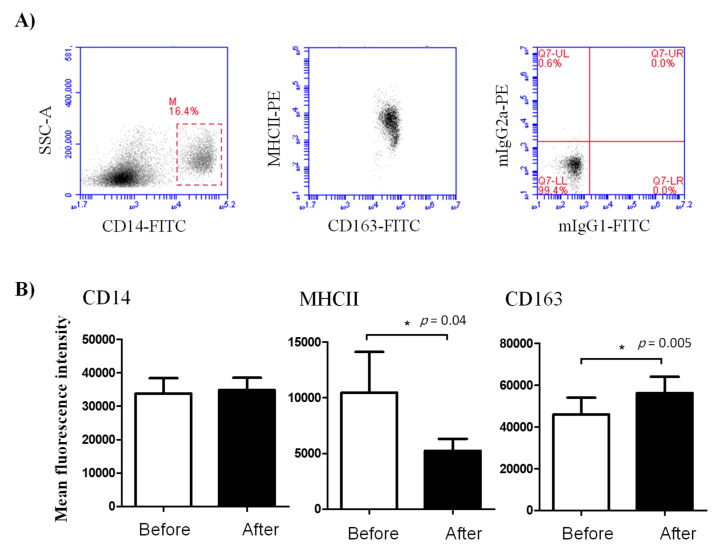
Ex vivo analysis of the abundance of the cell surface molecules CD14, MHC-II, and CD163 on camel blood monocytes. Leukocytes were labeled with mAbs to the cell antigens CD14, MHCII, and CD163, and the cell membrane fluorescence was measured on the flow cytometer. (**A**) After the identification of monocytes, based on their positive staining with CD14, the expression levels (MFI) of CD14, MHC-II, and CD163 were estimated for samples collected before and after treatment with lornoxicam. (**B**) Differences between the means were considered significant (*) if *p* < 0.05.

The expression density (mean fluorescence intensity, MFI) of the cell surface antigens CD14, CD163, and MHC-II on blood monocytes was significantly changed after treatment with lornoxicam (Figure 3). In comparison to their basic MFI values (before treatment), the expression density of MHC-II molecules on blood monocytes was significantly (*p* < 0.05) decreased after treatment with lornoxicam (MFI= 5243.5 ± 1063.5 versus 10450.9 ± 3654.2 before treatment). In contrast to this, monocytes significantly increased their CD163 expression after treatment with lornoxicam (MFI= 56333.1 ± 7710.0 versus 45978.9 ± 8041.6 before treatment). The expression density of CD14 on monocytes did not show a significant change after treatment (Figure 3).

### 3.4. Lornoxicam Injection Reduces the Phagocytosis and ROS Generation Capacity of Blood Phagocytes

**Figure 4:**
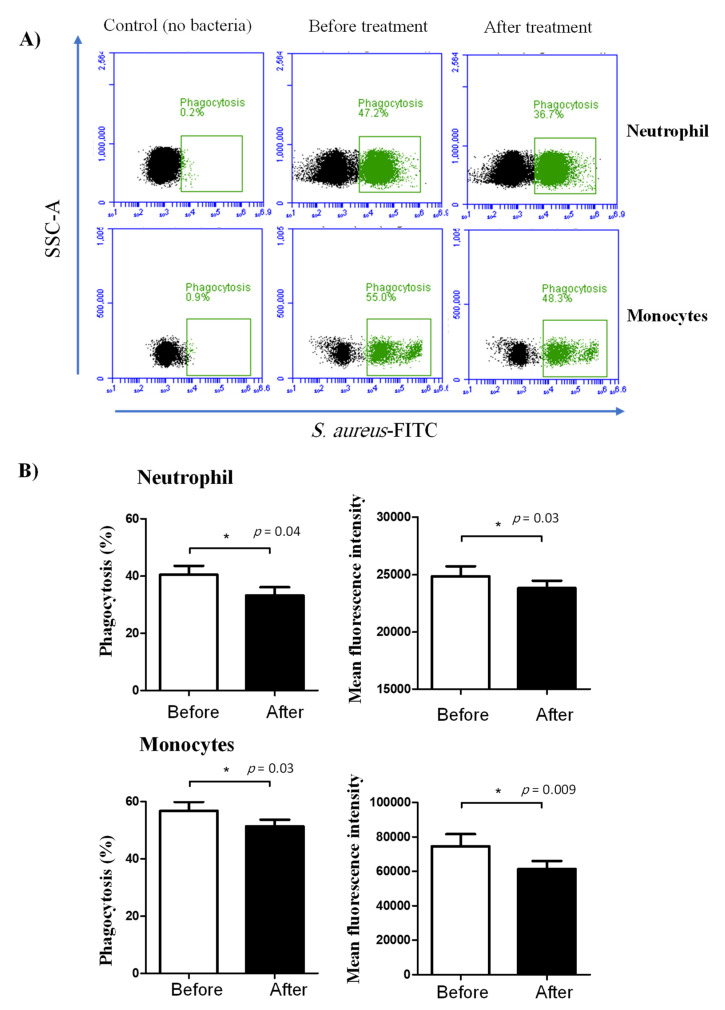
Ex vivo analysis of the phagocytosis activity of camel neutrophils and monocytes. Separated leukocytes were stimulated with FITC-labeled bacteria (*S. aureus)* and the immunofluorescence signals were detected by flow cytometry. (**A**) After gating on neutrophils and monocytes, the fraction and the MFI of phagocytosing cells were determined based on the increased green fluorescence in FL-1. (**B**) Data were presented for neutrophils and monocytes from camels before and after treatment with lornoxicam and were presented as means ± SEM. Differences between the means were considered significant (*) if *p* < 0.05.

The phagocytosis activity of blood neutrophils and monocytes was negatively affected by lornoxicam (Figure 4). The percentage of cells that phagocytized bacteria was significantly (*p* < 0.05) reduced for both neutrophils (33.3 ± 2.9 versus 40.5 ± 3.0% before treatment) and monocytes (51.4 ± 2.3 versus 56.8 ± 3.1% before treatment) in the blood of camels after treatment with lornoxicam when compared to their basic values (Figure 4). This was also the case for the phagocytosis capacity, which was estimated by the MFI of phagocytosing cells, indicating the relative amount of bacteria phagocytosed by each cell, which was significantly (*p* < 0.05) lower for both neutrophils and monocytes after treatment than before treatment with lornoxicam.

**Figure 5:**
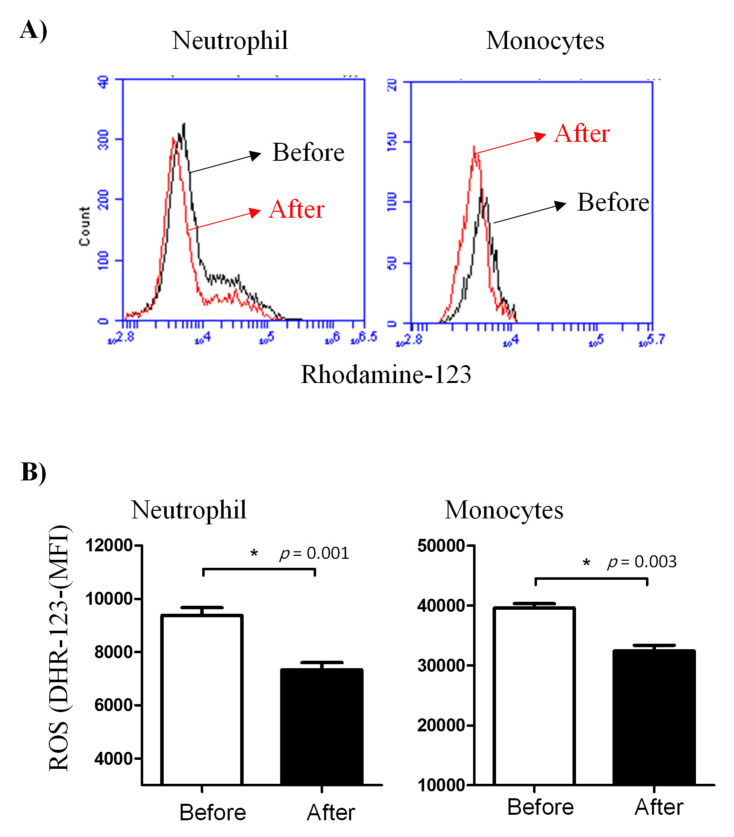
Ex vivo measurement of the ROS generation activity of camel leukocytes by flow cytometry. Leukocytes were isolated from the blood before and after administration of camels with lornoxicam and the separated cells were stimulated with heat-killed bacteria. (**A**) The ROS activity of camel leukocytes was estimated by increased green fluorescence due to the generation of rhodamine-123. (**B**) The MFI (mean ± SEM) of gated cells was presented for samples collected before and after treatment with lornoxicam. Differences between the means were considered significant (*) if *p* < 0.05.

Similarly, treatment with lornoxicam negatively affected ROS production in neutrophils and monocytes after bacterial in vitro stimulation. In comparison to their ROS production activity before treatment, neutrophils (DHR MFI= 7330.3 ± 272.8 versus 9376.1 ± 297.0 before treatment) and monocytes (DHR MFI= 32392.2 ± 959.5 versus 39594.5 ± 717.0 before treatment) collected from the animals after treatment with lornoxicam showed significantly (*p* < 0.05) lower ROS values (Figure 5).

### 3.5. Impact of In Vitro Incubation with Lornoxicam on Cell Vitality of Camel Leukocytes

**Figure 6:**
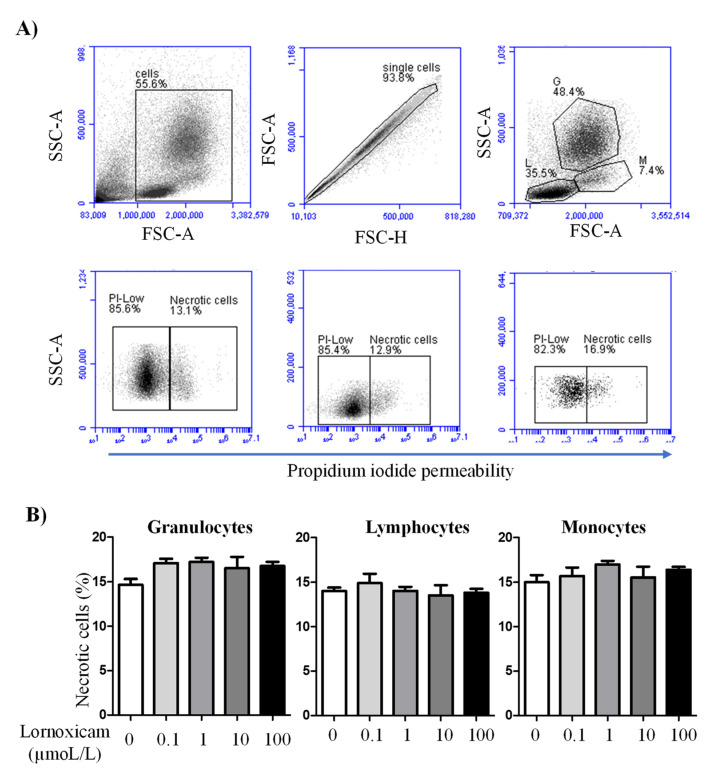
The impact of in vitro incubation with lornoxicam on camel leukocytes cell necrosis. Leukocytes were labeled with propidium iodide (PI) and PI-uptake versus exclusion was measured by flow cytometry. (**A**) Gating strategy for the measurement of leukocytes cell necrosis. In a FSC-A/SSC-A density plot, cell debris was excluded by gating on leukocytes according to their FSC and SSC properties. Within singlets (as identified in a FSC-H/FSC-A density plot), granulocytes (G), lymphocytes (L), and monocytes (M) were gated according to the cell-specific FSC and SSC characteristics. Viable (PI^low^) cells and necrotic (PI^high^ cells) were identified in a SSC-A/PI density plot. (**B**) The percentages of necrotic cells within the whole population of granulocytes, lymphocytes, and monocytes were presented as mean ± SEM. The differences between the treatments were considered significant if the *p* value was less than 0.05 (one-way ANOVA; repeated measures).

In vitro incubation of camel blood with increasing concentrations of lornoxicam for 6 h did not significantly (*p* > 0.05) change the percentage of necrotic cells (propidium iodide-permeable cells) within the whole cell population of camel granulocytes, lymphocytes, or monocytes (Figure 6A,B).

**Figure 7:**
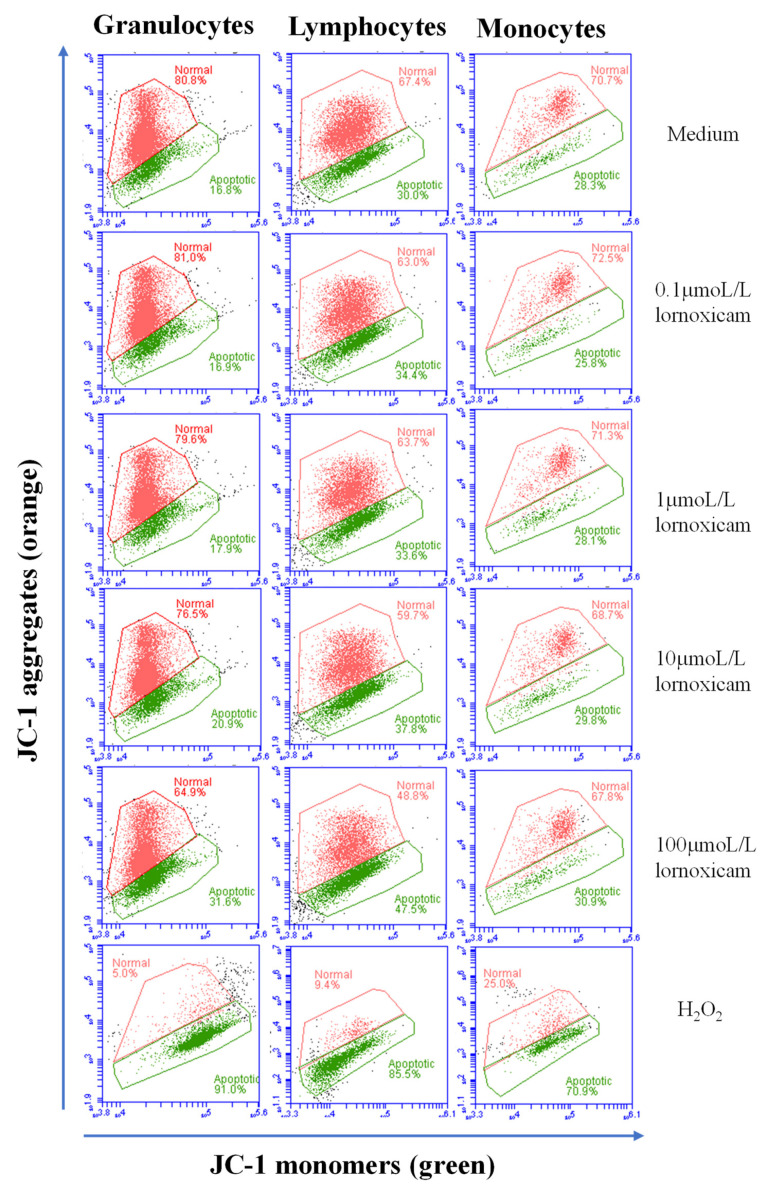
Analysis of leukocytes cell apoptosis using flow cytometry. Leukocytes were loaded with the JC-1 dye and the cell populations of granulocytes, lymphocytes, and monocytes were identified within singular cells based on cell type-specific FSC and SSC properties. The percentage of cells with orange JC-1 aggregates (non-apoptotic cells with increased fluorescence in FL-2) and cells with green JC-1 monomers (apoptotic cells with increased fluorescence in FL-1) were estimated for cells in medium and those collected from blood incubated with lornoxicam in vitro. Hydrogen peroxide (H_2_O_2_) has been used to establish a positive control of apoptosis.

**Figure 8:**
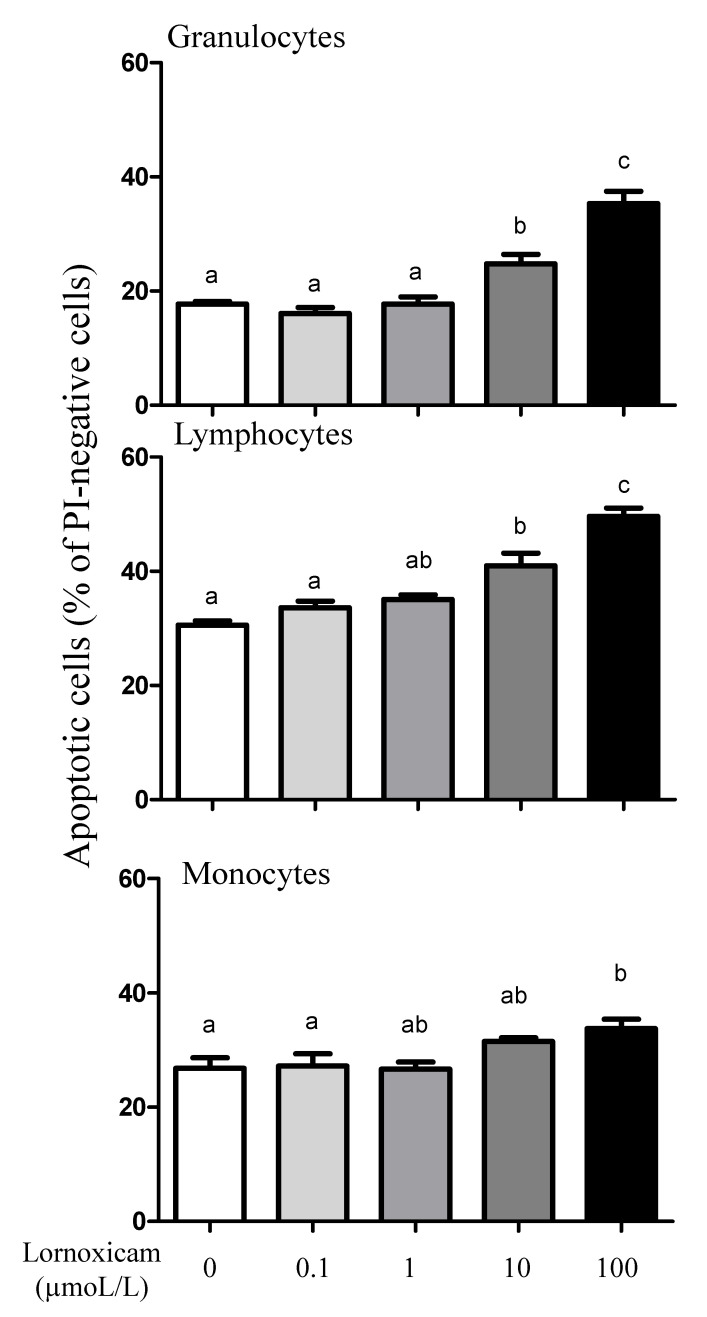
The impact of in vitro incubation with lornoxicam on leukocytes cell apoptosis. Leukocytes were separated from camel blood after incubation with different concentrations of lornoxicam in vitro. Leukocytes were labeled with the JC-1 dye and cell fluorescence signals were measured by the flow cytometer. The fraction of apoptotic cells within the whole populations of granulocytes, lymphocytes, and monocytes was calculated. Significant differences between the groups were indicated by different lower case letters (One-way ANOVA, repeated measures (*p* < 0.05)).

The analysis of cell apoptosis, however, revealed lornoxicam-induced changes in the percentage of apoptotic leukocytes (cells containing JC-1 monomers) (Figure 7). The percentage of apoptotic granulocytes and lymphocytes increased significantly (*p* < 0.05) in whole blood incubated with high concentrations of 10 and 100 µmol/L of lornoxicam. For granulocytes, the differences were significant between the concentrations 10 and 100 µmol/L and also between each of them and all other concentrations. For lymphocytes, the differences were significant between the concentrations 10 and 100 µmol/L and also between each of them and all other concentrations except the 1 µmol/L concentration. For monocytes, however, only incubation with 100 µmol/L of lornoxicam resulted in a higher percentage (*p* < 0.05) of apoptotic monocytes (Figure 8). There was no significant change (*p* > 0.05) in the percentage of apoptotic cells after incubation with lornoxicam in the concentrations 0.1 or 1 µmol/L (Figure 8).

### 3.6. Impact of In Vitro Incubation with Lornoxicam on Bacterial Phagocytosis and ROS Production Capacity of Camel Granulocytes and Monocytes

**Figure 9:**
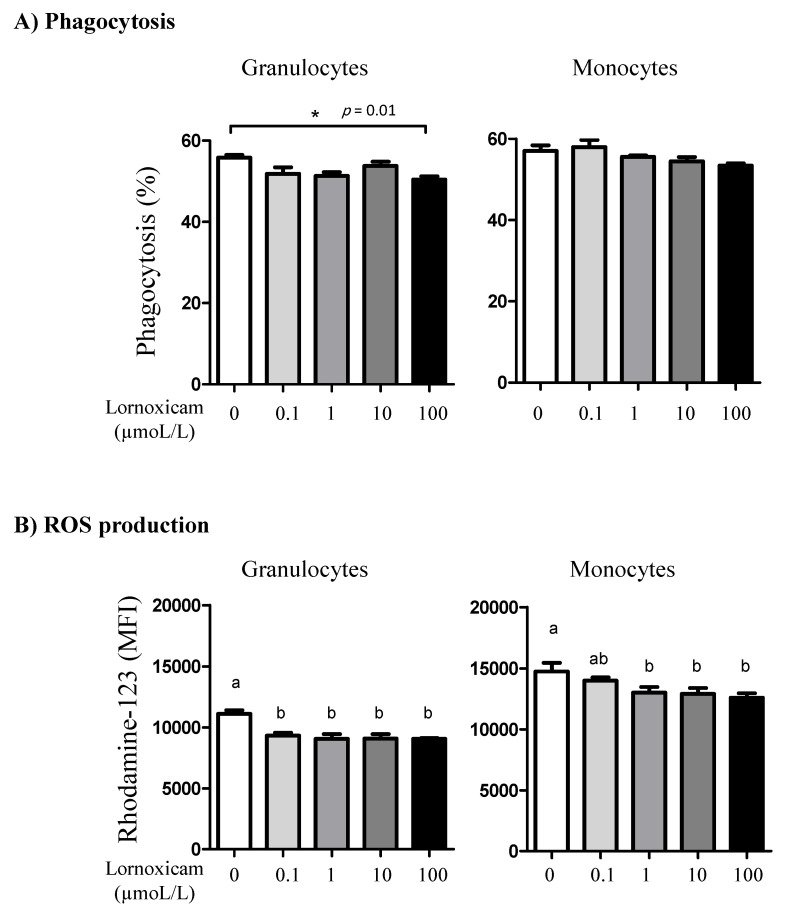
The impact of in vitro incubation with lornoxicam on phagocytosis and ROS production by camel granulocytes and monocytes. (**A**) Leukocytes were stimulated with FITC-labeled bacteria and the percentage of cells with ingested bacteria was determined by flow cytometry. * indicates significant differences between the means. (**B**) Impact of in vitro incubation with lornoxicam on ROS production by *S. aureus*-stimulated camel granulocytes and monocytes. Leukocytes were incubated with killed bacteria and dihydrorohdamin-123 (DHR-123) dye. Statistically significant differences between the treatments were measured using the one-way ANOVA (different lower case letters indicate a *p* < 0.05).

While the phagocytosis activity of monocytes did not show a significant change (*p* > 0.05) after in vitro incubation of camel blood with any lornoxicam concentration, only incubation with 100 µmol/L of lornoxicam resulted in a lower (*p* < 0.05) percentage of phagocytosis-positive granulocytes (Figure 9A). *S. aureus*-induced ROS production in granulocytes was significantly lower in blood incubated with lornoxicam at any concentration (0.1, 1, 10, and 100 µmol/L). For monocytes, a similar inhibitory effect (*p* < 0.05) of lornoxicam on ROS production was detected in blood incubated with the concentrations 1, 10, and 100 µmol/L (Figure 9B).

## 4. Discussion

Lornoxicam, a member of the oxicams group, is a nonsteroidal anti-inflammatory drug (NSAID) with potent anti-inflammatory, anti-pyretic, and analgesic effects. [12,13,14,15,16]. Oxicams, like all other NSAIDs, have also shown the potential for several adverse effects [21]. The improved tolerance of lornoxicam due to the relatively shorter elimination half-life in comparison to other members of the oxicams makes its application appear advantageous in the management of pain and inflammation in racing dromedary camels. As no studies were conducted on possible modulatory or suppressive potential of lornoxicam on the camel immune system, the current study aimed to investigate the ex vivo and in vitro effect of lornoxicam on some phenotypic and functional properties of camel blood leukocytes.

The injection of camels with a single dose of lornoxicam resulted in a significant decrease in the total leukocyte count with reduced numbers of all leukocyte populations, including neutrophils, eosinophils, monocytes, and lymphocytes. The in vitro pro-apoptotic effect of lornoxicam on camel leukocytes at concentrations that can be easily reached in plasma after the administration of lornoxicam in vivo indicates a role for lornoxicam-induced cell death in the reduced leukocyte numbers in lornoxicam-injected camels. A similar detrimental effect on cell viability was reported in bovine mammary epithelial cells treated with meloxicam [31]. Cell apoptosis is mainly regulated by caspases, a family of cysteine proteases, including caspase-2, -8, -9, -10, -11, and -12. In an in vitro model, incubation of canine kidney cells with meloxicam caused activation of caspase-9/-3 and release of cytochrome c, indicating the induction programmed cell death. Further studies are required to see, whether the pro-apoptotic effect of lornoxicam is mediated by the activation of caspases in camel leukocytes. As the number of leukocytes in the blood is additionally regulated by their production rate in the bone marrow, we cannot exclude a possible inhibitory effect of lornoxicam on the hematopoiesis in the bone marrow. In cattle with transport-induced stress, a stress alleviating effect of meloxicam has been suggested by reducing the percentage of circulating neutrophils and monocytes [11]. The general leukocytopenia in camels injected with lornoxicam, however, indicates a suppressive effect rather than a selective modulatory effect on the immune system of camels. This is also supported by the reduced cell numbers of all subsets within the lymphocyte population, including CD4+ T cells, γδ T cells, and B cells, indicating a general inhibitory effect of lornoxicam on the humoral and cell-mediated immunity. Furthermore, the elevated neutrophil to lymphocyte ratio, which is widely accepted as indicative for impaired immune cell function [32,33], in lornoxicam-treated camels also indicates a suppressive impact of lornoxicam on the camel immune system. This is, however, in contrast to the reported immuno-stimulating effect of meloxicam, another member of the oxicams group, on the proliferation of bovine natural killer (NK) cells in vitro [34].

The modulatory effect of lornoxicam on the phenotype of blood monocytes was characterized by a marked decrease in the expression density of MHC class II molecules with a significant increase in CD163 expression, a phenotype associated with an anti-inflammatory functional subtype of macrophages [23,35,36,37,38,39,40]. In LPS-stimulated human monocytic cells (THP-1), lornoxicam showed a marked inhibition of IL-6 and nitric oxide production (IC50 54 μM) [17].

Subsequently, we tested whether the effects of lornoxicam extend to the functional properties of camel blood phagocytes. The ex vivo analysis of the capacity of neutrophils and monocytes to phagocytize bacteria and to produce ROS upon bacterial stimulation revealed a significant inhibitory effect of lornoxicam on the antimicrobial activity of both neutrophils and monocytes. The inhibitory effect of in vitro incubation with lornoxicam (100 µmol/L) on phagocytosis by granulocytes and ROS production by granulocytes and monocytes (by almost all lornoxicam concentrations) supports the ex vivo findings. Incubation of humans neutrophils with lornoxicam at concentrations similar to those used in the present study resulted in the inhibition of their in vitro cell migration [41]. A similar inhibitory effect on ROS production was reported for human neutrophils treated with meloxicam [42]. In bovine calves with enzootic bronchopneumonia, treatment with meloxicam in combination with oxytetracycline, however, did not affect the phagocytic activity of blood phagocytes [43]. Several studies reported the potential of NSAID for impairing various functions of the immune system by disturbing the functions of immune-competent cells [44,45,46]. The effects of NSAID on immune function, however, vary by compound and species. Mild immune suppressive effect of lornoxicam has been reported in humans when compared to other analgesics such as morphine and tramadol [21]. Furthermore, Memis et al. reported no effect of intravenous lornoxicam injection on the level of several cytokines in immune-compromised sepsis patients [47]. Phagocytosis of bacterial pathogens by the phagocytes, neutrophils and monocytes, and their subsequent killing by ROS-mediated mechanisms are key effector functions with a major role in the innate immune defense against bacteria [48]. The inhibitory effect of lornoxicam on the phagocytosis and ROS production of monocytes and neutrophils suggests its potential impairment of innate immunity to bacterial pathogens in camel. Similarly, the observed decrease in several immune cell types in lornoxicam-treated camels indicates a general inhibitory effect on the camel cellular immune system. However, further studies are required to investigate the clinical relevance of the lornoxicam-induced changes in immune cell composition and function in camels.

The anti-inflammatory activity of lornoxicam is mainly related to its inhibitory effect on prostaglandin and thromboxane synthesis through the inhibition of both COX-1 and COX-2, inducible nitric oxide synthase (iNOS), and interleukin 6 production [17]. Whether the lornoxicam-induced changes in leukocyte composition and function are mediated by the same mechanisms, still to be investigated.

## 5. Conclusions

In summary, the present study found evidence that a single dose of intravenously administrated lornoxicam, at 0.13 mg/kg, induces, six hours after injection, a significant change in the camel leukogram which is characterized by reduced cell numbers of all leukocyte subpopulations. In vitro analysis of cell vitality revealed a pro-apoptotic effect of lornoxicam on camel leukocytes, which may be responsible for the lornoxicam-induced leukocytopenia in vivo. Functionally, lornoxicam elicited inhibitory effects on the antimicrobial capacity of the blood phagocytes, monocytes and neutrophils. Furthermore, lornoxicam induced an anti-inflammatory phenotype of monocytes, characterized by reduced expression of MHC class II molecules and increased expression of CD163 molecules. The current study identified for the first time inhibitory effects of the COX-inhibitor lornoxicam on phenotype and function of camel blood immune cells and recommend considering these effects when using lornoxicam in camel medicine

## Figures and Tables

**Table 1:** List of antibodies.

Antigen	Antibody Clone	Labeling	Source	Isotype
CD14	TÜK4	-	WSU	Mouse IgG1
MHCII	TH81A5	-	Kingfisher	Mouse IgG2a
CD163	LND68A	-	WSU	Mouse IgG1
CD4	GC50A1	-	WSU	Mouse IgM
WC1	BAQ128A	-	WSU	Mouse IgG1
Mouse IgM	poly	APC	Thermofisher	Goat IgG
Mouse IgG1	poly	FITC	Thermofisher	Goat IgG
Mouse IgG2a	poly	PE	Thermofisher	Goat IgG

CD: cluster of differentiation; Ig: immunoglobulin; MHC: major histocompatibility complex; APC: allophycocyanin; FITC: fluorescein isothiocyanate; PE: phycoerythrin; poly: polyclonal.

## Data Availability

The datasets used and/or analyzed during the current study are available from the corresponding author on reasonable request.
